# Cytokine Networks as Targets for Preventing and Controlling Chagas Heart Disease

**DOI:** 10.3390/pathogens12020171

**Published:** 2023-01-21

**Authors:** Carolina Cattoni Koh, Eula G. A. Neves, Thaiany Goulart de Souza-Silva, Ana Carolina Carvalho, Cecília Horta Ramalho Pinto, Alexsandro Sobreira Galdino, Kenneth J. Gollob, Walderez Ornelas Dutra

**Affiliations:** 1 Laboratório de Biologia das Interações Celulares, Departamento de Morfologia, Instituto de Ciências Biológicas, Universidade Federal de Minas Gerais, Belo Horizonte 31270-901, MG, Brazil; 2 Laboratório de Biotecnologia de Microrganismos, Universidade Federal de São João Del-Rei (UFSJ), Campus Centro Oeste, Divinópolis 35501-296, MG, Brazil; 3 Hospital Israelita Albert Einstein, São Paulo 05652-900, SP, Brazil; 4 Instituto Nacional de Ciências e Tecnologia em Doenças Tropicais, INCT-DT, Salvador 40110-160, BA, Brazil

**Keywords:** cytokines, immunoregulation, gene polymorphism, chagas disease, *Trypanosoma cruzi*

## Abstract

Chagas disease, a neglected disease caused by the protozoan *Trypanosoma cruzi*, is endemic in 21 Latin American countries, affecting 6–8 million people. Increasing numbers of Chagas disease cases have also been reported in non-endemic countries due to migration, contamination via blood transfusions or organ transplantation, characterizing Chagas as an emerging disease in such regions. While most individuals in the chronic phase of Chagas disease remain in an asymptomatic clinical form named indeterminate, approximately 30% of the patients develop a cardiomyopathy that is amongst the deadliest cardiopathies known. The clinical distinctions between the indeterminate and the cardiac clinical forms are associated with different immune responses mediated by innate and adaptive cells. In this review, we present a collection of studies focusing on the human disease, discussing several aspects that demonstrate the association between chemokines, cytokines, and cytotoxic molecules with the distinct clinical outcomes of human infection with *Trypanosoma cruzi*. In addition, we discuss the role of gene polymorphisms in the transcriptional control of these immunoregulatory molecules. Finally, we discuss the potential application of cytokine expression and gene polymorphisms as markers of susceptibility to developing the severe form of Chagas disease, and as targets for disease control.

## 1. Introduction

According to the World Health Organization (WHO), Chagas disease, a parasitic disease caused by infection with *Trypanosoma cruzi*, leads to approximately 14,000 deaths annually and is one of the main causes of sudden death, which often occurs in the most productive phase of the patient’s life. The disease is still considered a serious social and public health problem, despite the advances made in its control and prevention [1,2,3,4].

The main form of *T. cruzi* infection in endemic setting is via the contact with contaminated excreta from the invertebrate host, a triatomine of the Reduviidae family, that occurs during the vector’s blood meal. However, other forms of parasite transmission via blood transfusion, organ transplantation, congenital transmission, ingestion of contaminated food and, less frequently, laboratory accidents, may occur [5,6,7,8].

After infection, an acute phase of short duration is followed by the chronic phase of the disease, which can last for years or decades. The acute phase is characterized by local tissue degeneration and inflammatory changes due to high parasitemia [9,10]. In approximately 90% of cases, the clinical manifestations last from a few weeks to a few months, and spontaneously regress with a decrease in parasitemia. In most cases, the acute phase can be asymptomatic, or patients may display unspecific symptoms [11]. Lack of specific symptoms is one of the challenges in the early diagnosis of CD, which has implications for therapeutic interventions. Thus, finding new diagnostics, especially a point-of-care that can be applied in the field is a pressing matter. Regarding this effort, new approaches have recently been developed and have been extensively reviewed in a recent paper focused on Chagas disease diagnosis [12].

Around 70% of patients who progress to the chronic phase of CD remain asymptomatic, characterizing the indeterminate clinical form (IND). In these patients, the disease is only detected by positive results from at least two specific serological tests since the patients do not display any clinical signs or symptoms of the disease. Approximately 30% of chronic patients display digestive and/or cardiac alterations during the chronic phase, which may lead to death. Amongst the symptomatic forms, the cardiac clinical form has the highest morbidity and mortality rate [13,14]. The distinct clinical evolution in the chronic phase results from multifactorial mechanisms. For instance, parasite aspects, such as strain variability and tropism [15,16], and host aspects, such as age, sex, nutritional, socioeconomic factors, and immunological characteristics [17,18,19,20] are known to influence disease progression.

## 2. The Role of Cytokines in Immune Regulation during the Chronic Phase of Chagas Disease

**Figure 1:**
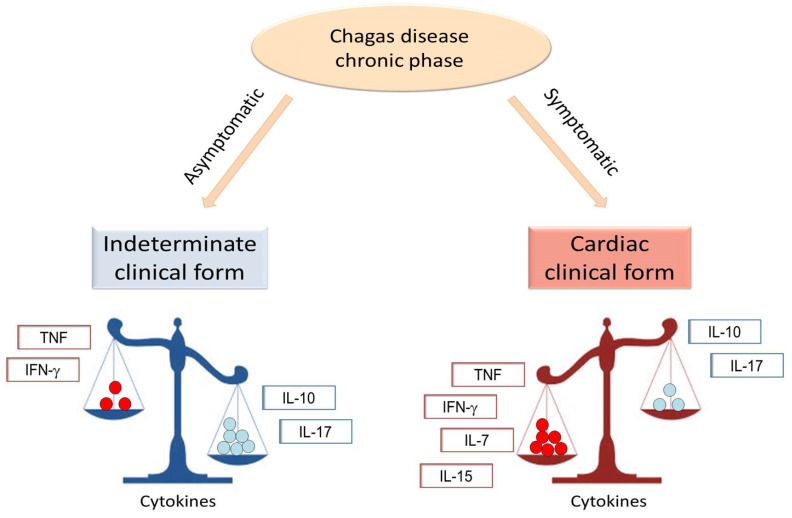
**Schematic representation of cytokine expression in the indeterminate and cardiac clinical forms of Chagas disease**. In the indeterminate clinical form, an increased expression of anti-inflammatory cytokines, such as IL-10 and IL-17 is observed. However, in the cardiac clinical form, the increased expression of pro-inflammatory cytokines, such as IFN-gamma and TNF, favor the establishment of the inflammatory environment. Cytokines, such as IL-7 and IL-15, have been associated with the cardiac clinical form.

Cytokines are molecules actively involved in immune regulation during the chronic phase of Chagas disease (Figure 1) and, although their role in cell activation is essential for infection control, it can contribute to myocardial dysfunction [21,22]. While a high expression of mRNA that code for pro- and anti-inflammatory cytokines has been observed in mononuclear cells from patients chronically infected with *T. cruzi* [23], studies performed by several groups showed that the production of such cytokines was distinct in patients with different clinical forms of the disease (Table 1). While the role of these cytokines provides insight as to the mechanism by which they act, more studies are needed to address this issue. The release of pro-inflammatory cytokines in the plasma, as well as their expression by peripheral blood mononuclear cells (PBMC) and by the myocardium, characterize the intense inflammation and Th-1-like response that is observed in patients with chronic Chagas cardiomyopathy (CCC) [24,25,26,27,28,29,30,31,32,33]. This exuberant inflammatory immune activation observed in CCC patients is high, even if compared to inflammatory cardiomyopathies of other etiologies, such as rheumatic heart disease and idiopathic cardiomyopathy [34,35]. On the other hand, anti-inflammatory cytokines are predominant in the immune milieu of IND patients, despite the concurrent production of anti- and pro-inflammatory cytokines observed in these patients [36].

Cytokines can be produced by a plethora of distinct cell populations, and identifying the source of the cytokines, as well as the antigens that induce their expression may guide immunotherapeutic interventions. The functional analysis of human monocytes after in vitro infection with *T. cruzi* trypomastigotes showed that, in CCC patients, these cells display high expression of TNF. Conversely, monocytes from IND patients are compromised with the production of IL-10 [18,51]. In addition, classical (CD14++CD16-) and inflammatory (CD14++CD16+) monocytes are positively correlated with IL-6 production, described as a biomarker of cardiac failure and severity in CCC patients [44,45,65]. Although monocytes are an essential source of cytokine in chronic Chagas disease, they are also crucial for antigenic presentation to T lymphocytes, which are highly activated in the chronic phase of infection [66].

The presence of circulating CD4+ T cells that produce high levels of IFN-gamma and low IL-10/IFN-gamma ratio reported in CCC patients confirms the Th1 profile associated with intense inflammation. Menezes et al. (2004) demonstrated a positive correlation between the frequency of CD4+ TNF+ cells and CD4+CD28- cells in CCC patients. Alternatively, CD4+ CD28- cells were positively correlated with the frequency of CD4+IL-10+ cells in IND patients, suggesting the involvement of distinct mechanisms of immunoregulation between the symptomatic and asymptomatic clinical forms of the disease [67].

This immunological polarization is also observed among other cytokine-producing cell subpopulations. T cells that do not express the co-receptors CD4 and CD8, named double-negative (DN) T cells, have been described as potent cytokine producers in many diseases [68]. After in vitro stimulation with *T. cruzi* trypomastigotes, DN T cells expressing the T-cell receptor (TCR) alpha-beta from CCC patients show high expression of inflammatory cytokines (IFN-gamma, TNF). On the other hand, the TCR gamma-delta+ DN T cells are involved with high IL-10 expression and better cardiac function in IND patients [50]. Interestingly, stimulation of TCR gamma-delta+ DN T cells with a glycolipid-rich fraction of *T. cruzi* increases the IFN-gamma-mediated inflammatory profile in CCC patients [69]. Conversely, blocking the in vitro activation of DN T cells from CCC patients reduces the frequency of IFN-gamma in TCR gamma-delta+ DN T cells and increases the frequency of IL-10 in effector memory TCR gamma-delta+ DN T cells, favoring the establishment of a less inflammatory environment [70,71]. Furthermore, in IND patients, central memory DN T cells show a balanced immune response characterized by the co-expression of circulating IL-10+ IFN+ cells [70].

In addition to the DN T cells, another minority circulating cell population that displays a dichotomic cytokine profile in Chagas disease associated with distinct clinical forms are the B1 B cells. These cells represent a subset of B cells involved with the production of natural and auto-reactive antibodies [72,73], as well as significant IL-10 expression [74]. Previous studies have shown that the frequency of B1 B cells is increased in chronic Chagas disease patients [66], and that the frequency of these cells is restored to normal levels in Chagas patients submitted to successful chemotherapy [75]. It has been shown that B-1 B cells from Chagas patients respond to parasite antigens [75], and are preferentially activated by a component of this antigen that is enriched in proteins, but not lipids and carbohydrates [41]. In addition, these activated B1 B cells produce different cytokine profiles depending on whether they come from IND or CCC patients: while activated TNF-producing B1 B cells are observed in cultures of PBMC from CCC-stimulated protein-rich parasite-derived antigens, a concomitant production of TNF and IL-10 was observed in cultures of PBMC from IND under the same condition [41]. Importantly, these potentially regulatory activated B1 B cells are correlated with better cardiac function in Chagas disease [41]. Moreover, the presence of regulatory B2 B-cells, the great majority of antibody-producing B-cells, have been correlated with the indeterminate form of Chagas disease [56]. These data support the hypothesis that a balanced anti-inflammatory/pro-inflammatory profile in IND patients, established with the involvement of different cell subpopulations, prevents tissue damage and progression to symptomatic forms of the disease.

The systemic and cellular inflammatory profile observed in CCC patients is mirrored by the expression of cytokines in the cardiac tissue [28,31,37]. This scenario contributes to cardiomyocyte apoptosis and, consequently, to heart tissue damage and remodeling, which involves fibrosis [39,64,76,77]. In addition, these immune mediators may contribute to cellular recruitment and survival of inflammatory T cells in the myocardium [42,78].

Therefore, understanding the cytokine networks involved with the pathogenic immune response in CCC and with the protection in IND patients may elucidate important immunological targets to prevent progression to symptomatic forms of the disease, as well as control the intense inflammatory response associated with the cardiac pathology.

## 3. Cell Cytotoxicity in the Pathogenesis of Chronic Chagas Cardiomyopathy (CCC)

CCC is characterized by the development of myocarditis, tissue fibrosis, and cardiac hypertrophy [79,80]. The inflammatory infiltrates present in the myocardium of CCC patients are composed mainly of CD8+ T cells, which reinforces the role of cytotoxic activity in cardiac tissue damage [81,82,83,84].

CD8+ T cells release cytolytic granules, composed of perforin and granzymes, toward the target cells. Perforin forms pores in cell membranes and allows the entry of granzymes that activate caspase pathways. This can result in apoptosis induction of target cells infected by intracellular microorganisms, such as *T. cruzi* [83,84,85].

The presence of lysosomal-associated membrane protein-1 (LAMP-1 or CD107a) on the surface of cytolytic cells characterizes the process of cell degranulation and the effector immune response in infectious diseases [86]. In 2015, Lasso et al. showed that CD8+ T cells from patients with CCC displayed an increase in the expression of CD107a/b, perforins, and granzyme B when stimulated with *T. cruzi* antigens and the KMP-11 recombinant protein, indicating that these cells increase their cytotoxic potential during chronic infection [87]. Reis et al. (1993) had previously reported the expression of CD8+ granzyme A+ cells in myocardial lesions of CCC patients, supporting the idea that cell cytotoxic mechanisms mediate cardiomyocyte destruction [28].

CD4+ cells have also been described as potentially cytotoxic in CD. A positive correlation was demonstrated between CD4+ cells expressing the variable region beta 5 chain (Vβ5) of the T-cell receptor (TCR), and the expression of granzyme A in CCC patients [88]. Furthermore, in 2012, Keesen et al. (2012) demonstrated an increased frequency of granzyme B and CD107 on circulating CD4+ T cells from IND and CCC patients, when compared to healthy donors. Interestingly, the authors showed an association between the expression of granzyme B and CD107a with the memory marker CD45RO in CD4+ cells from patients in the IND group after stimulation with *T. cruzi* antigen. This indicates that the expression of cytotoxic markers by this cell subpopulation may be necessary to control the immune response in asymptomatic patients [89]. However, it is worth speculating which factors are associated with the immunological imbalance that leads to cellular cytotoxicity in CCC patients. It is also important to highlight that the frequency of CD4+ granzyme A+ T cells is higher in patients with CCC compared to idiopathic dilated cardiomyopathy (IDC) [35]. Furthermore, the potentially cytotoxic CD4+CD28 cells are increased in patients with chronic Chagas disease, when compared to uninfected individuals [90,91].

The mechanisms of cell recruitment from the blood to cardiac tissue are not fully understood. However, it is known that the expression of adhesion molecules and chemokines may contribute to the migration of cell subpopulations with cytotoxic and inflammatory potential. In 1996, Laucella and collaborators demonstrated an association between serum s-p-selectin and the severity of chronic Chagas disease [92]. Furthermore, the upregulation of ICAM-1, VCAM-1, and LFA-1 in the myocardium of CCC patients points to the role of T-cell recruitment and inflammation in cardiac pathogenesis [92].

The engagement of adhesion molecules and chemokines are essential orchestrators of leukocyte migration. In 2004, Talvani et al. demonstrated that the release of CCL2 in plasma and the supernatant of PBMC from patients infected with *T. cruzi* is higher in those with severe cardiac impairment than in the group with mild heart disease [32]. Furthermore, samples from CCC patients showed elevated plasma levels of CCL3 and CCL4 when compared to patients with digestive disorders (32). The serum levels of chemokines CXCL10 and CXCL9 were increased in Chagas patients when compared to uninfected individuals [46]. Moreover, the plasma of CCC patients, when compared to healthy donors, showed increased levels of different systemic chemokines involved with the cellular recruitment of leukocytes and granulocytes [34]. This reinforces the role of strong immune activation and cellular recruitment in the pathogenesis of CD.

In 2005, Gomes et al. reported that CCC displayed an increased frequency of CXCR3 and/or CCR5 expression by CD4+ and CD8+ T cells. Interestingly, these cells produce the inflammatory cytokines IFN-gamma and TNF [64]. Additionally, when studying the progressive evolution of CCC, Roffe et al., in 2019, demonstrated a gradual increase in the expression of effector cells expressing CCR5 [62]. Consistent with these data, other studies showed that circulating and infiltrating mononuclear cells in the myocardial tissue of CCC patients present the expression of CXCR3, CCR5, CXCL9, and CCL5 [60,93].

**Figure 2:**
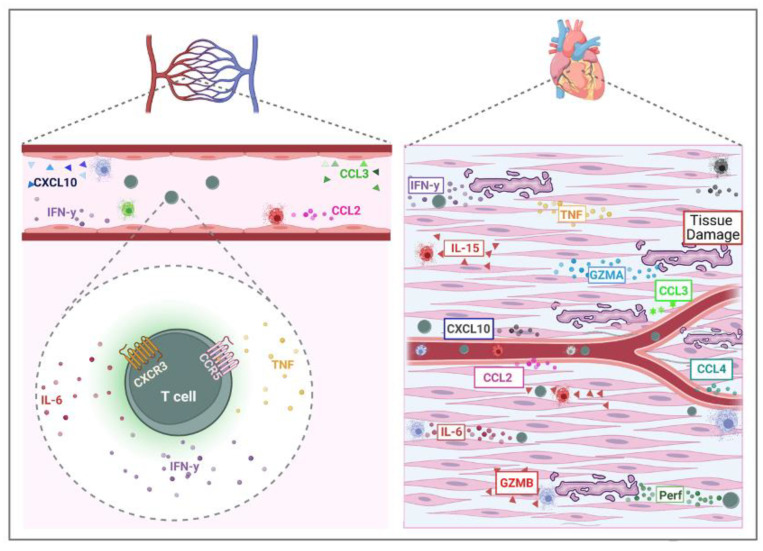
**Cytotoxic and inflammatory immune response in chronic Chagas cardiomyopathy.** T cells mediate cytotoxicity in chronic Chagas cardiomyopathy. These cells are recruited to the heart by adhesion molecules and chemokines, and can release inflammatory cytokines and cytotoxic molecules, such as granzymes and perforins, that contribute to cardiac tissue damage, fibrosis, and disease severity (Designed with Biorender).

Importantly, circulating CD8+ T cells from CCC patients show higher co-expression of CCR5 and cMet, chemotactic receptors involved in T-cell cardiac tropism, compared to IDC patients. Furthermore, the association between CD8+ CCR5+ cMet+ cells with IFN-gamma and the Eomes transcription factor highlights the potential of these cells for the recruitment of inflammatory and cytotoxic molecules capable of mediating cardiac tissue damage in CCC patients [35]. These findings demonstrate the participation of chemokines and their receptors in mediating the recruitment of immune cells with inflammatory and cytotoxic activity in the myocardium of CCC patients, which can induce tissue destruction and cardiac pathology (Figure 2). However, studies in this area are still scarce and further analysis evaluating the association of these molecules with cytotoxic activity may elucidate the mechanisms involved in cardiac tissue destruction in CD.

## 4. Control of Cytokine Expression

Factors that lead IND patients to develop CCC are still unknown. However, studies indicate that mechanisms of gene regulation and cytokine-mediated signaling may be involved in the susceptibility to developing cardiomyopathy [94]. Amongst these mechanisms, the presence of single nucleotide polymorphisms (SNPs) and alteration of transcription factors emerges as important.

SNPs are changes in the DNA that occur in at least 1% of a given population, where a nucleotide is exchanged for another. SNPs can occur in every region of the genome, such as introns, exons, promoters, enhancers, or in between genes [95,96]. Some gene polymorphisms can lead to changes in the levels of expression of the final protein, as well as alterations in its functions, and are, thus, classified as functional polymorphisms.

Several studies have demonstrated that genotypic alterations can influence the production of biological molecules in CD [97,98,99], suggesting that SNPs may be related to susceptibility to CD and/or to the establishment of severe CCC. SNPs are often used as biological markers to locate genes that are associated with the disease. For over 30 years, numerous studies related to polymorphism in genes that encode cytokines, chemokines, multiple receptors, and important molecules in the antigens presentation (MHC) have been published. The selected studies are presented in Table 2 displaying the molecules by their effector characteristics. While this data show the implication of polymorphisms in disease outcomes, it is important to emphasize that some characteristics may vary between populations causing different implications and results according to the sample studied [100].

Despite the number of studies performed related to polymorphism association with disease susceptibility and severity, there are still important candidates that have not been evaluated. For example, studies related to CD and polymorphism in genes encoding cytotoxic molecules, critical for pathology, are lacking. Until now, few genome-wide association studies (GWAS) have been conducted, and there are no GWAS studies linking SNPs and changes in immune response in Chagas disease. Thus, studies on different molecules associated with CD and the assessment of their potential use as genetic biomarkers are necessary. In addition to gene polymorphisms, epigenetic alterations and activation of transcription factors are also important in controlling the expression and function of cytokines and other immune proteins.

Epigenetics is characterized by reversible changes in the expression and activity of one or more genes, without modifying the DNA sequence. Epigenetic changes occur by chemical changes in DNA bases which can result in changes in chromosome structure and packaging, maintaining the same nucleotide sequence [143]. The main epigenetic mechanisms are DNA methylation, histone modification, and non-coding RNA expression. DNA methylation consists of the addition of a methyl group (-Ch3) at the 5’ position of the DNA cytosine (C) and it occurs most frequently in cytosines that are immediately followed by a guanine (G). Regions rich in this sequence are called CpG islands and are commonly located in the promoter region of genes. Thus, methylation is associated with gene silencing, blocking gene transcription [144,145,146].

Another epigenetic mechanism is post-translational modifications in histone proteins. Histones are proteins that DNA binds around that are important for the condensation of the genetic material in the nucleus, regulating gene expression [147]. These modifications include acetylation (addition of an acetyl group), methylation (addition of a methyl group), phosphorylation (inclusion of a negative phosphate group to the histone tail), and ubiquitylation, which is the annexation of a large molecule of ubiquitin to lysine residues. Each of these changes interferes with DNA-histone interactions and can activate or block gene transcription [147,148,149,150].

Another class of epigenetic mechanisms is non-coding RNAs (ncRNAs). The ncRNAs are transcribed but not translated into proteins. Among ncRNAs, we can highlight the microRNAs (miRNAs) that are small, endogenous, and participate in the post-transcriptional regulation of cell signaling pathways [151,152]. miRNAs bind to complementary mRNAs, regulating expression through their destruction or the avoidance of protein translation (89). Several studies demonstrate the influence of miRNAs in various biological processes such as cell death, cell proliferation/differentiation, and immune regulation [153,154].

There are a few studies in the literature on epigenetic mechanisms and CD. Among these mechanisms, the most studied are microRNAs. A lower expression of miR-1, miR-133a, miR-133b, miR-208a, and miR-208b microRNAs were identified in heart tissue samples of CCC patients when compared to the healthy control group [155]. In addition, it was observed that during the chronic phase of the disease, IND patients have overexpression of microRNA-208a in plasma, when compared to cardiac patients, which may be a possible biomarker of disease progression [156]. In 2019, Nonaka et al., showed that the microRNAs MiR-19a-3p, miR-21-5p, miR-29b-3p, miR-30a, and miR-199b were differentially expressed in patients with CCC when compared to the indeterminate form. Additionally, their expression presented a positive correlation with cardiac dysfunction and fibrosis, and a negative correlation with ejection fraction and left ventricular tension, suggesting a relationship between the expression of these microRNAs and disease progression [157]. The difficulty of obtaining human samples and the limitation in simulating in vitro a biological environment are factors that may explain why there are so few epigenetic studies in Chagas disease patients. Thus, experiments in animal models and in silico analysis are strategies used to better understand the role of miRNA in this disease.

In 2015, Navarro et al., infected C57BL/6 mice with Colombian *T. cruzi* strain, evaluated the expression of different miRNAs at 15, 30, and 45 days after infection, and identified which miRNAs were differentially expressed. They reported that an association was observed between changes in the QT interval and the expression of miRNAs. miR-20, miR-20b, miR-21, miR-142, miR-146a, miR-146b, miR-155, miR-182, miR203, miR-222 had an increased expression, while in miR-139, miR -145, miR-149, miR-322, miR-503, a reduction of expression was observed [158]. The same group, in 2017, evaluated the role of miRNAs in the regulation of transcriptional changes during the acute phase of *T. cruzi* infection in mice. Using the computational evaluation of the integrated genome-wide analysis of genes and miRNA expression changes, the authors predicted that several miRNAs are related to disease progression of arrhythmia, fibrosis, myocarditis, and hypertrophy of the heart and that miR-238-3p, miR-149-5p, miR-143-3p, miR-145-5p, and miR-486-5p are present in these four pathological changes [159]. It was observed that miR-21, miR-146a, and miR-155 were overexpressed in cardiac tissue and plasma of mice infected with *T. cruzi*, both in the chronic and acute phases. Of these microRNAs, only miR-146a was found to be expressed in both stages of the disease, thus emerging as a potential biomarker of infection [160]. The upregulation of miR-21 and collagen expression was observed in serum samples from CCC patients, cardiac tissue from infected mice, and tests in cardiac fibroblast culture. Thus, this specific microRNA would be a mediator involved in the pathogenesis of cardiac fibrosis, being a potential therapeutic target for CCC [161]. Furthermore, it was observed that the lack of miR-155 caused strong parasitic infection and decreased the survival of infected mice, and these showed a reduction in the production of IFN-gamma and TNF, which are pro-inflammatory cytokines [162].

**Figure 3:**
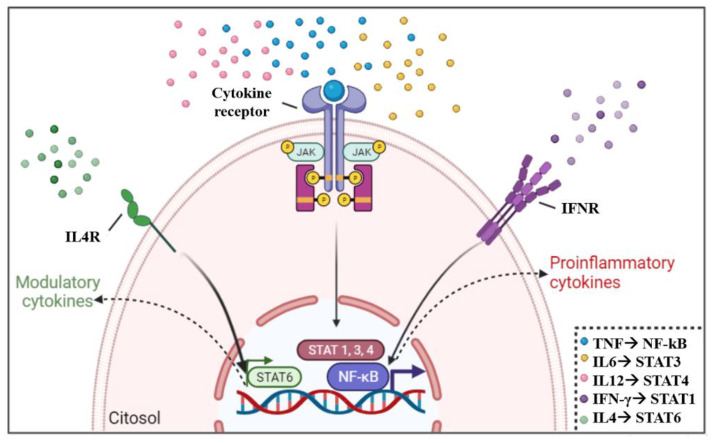
**Cytokine activation of STAT and association with Th1/Th2 development.** The engagement of inflammatory cytokines, such as IFN-gamma, IL6, IL12, and TNF, with their receptors favors the activation of transcription factors STAT1, STAT3, STAT4, and NF-kB, which contributes to the production of Th1 cytokines. While in modulatory environments, the presence of IL4 cytokine activates STAT6, which contributes to the production of Th2 cytokines. The association of STAT with cytokines (right corner of figure) emphasizes the main STAT associated with the cytokine, although other STAT may also be activated by the same cytokine (Designed with Biorender).

Cytokines bind to their receptors on the cell surface, leading to activation or inactivation of STATs and NF-kB, which are transcription factors capable of interfering with gene transcription. Target genes regulated by STATs are related to cell survival, growth, apoptosis, host defense, cell stress, and differentiation functions depending on the signaling pathway and target tissue. The NF-kB pathway plays a critical role in regulating the survival, activation, and differentiation of innate immune cells and inflammatory T cells [163,164]. For example, Th1 lymphocyte subpopulations respond to the binding of proinflammatory cytokines to their receptors, such as IFN-γ, IL-6, IL-12, and TNF, which will activate STATs 1, 3, 4, and NF-kB, respectively [39,164]. However, Th2 cells develop a regulatory response, mainly mediated by IL-4 binding, promoting STAT6 pathway activation [165]. Although not directly associated with Th1 and Th2 activation, STAT2 is critical for macrophage activation and type 1 interferon responses [166] and STAT5 is associated with cell proliferation and apoptosis [167]. Activation pathways of transcription factors by the engagement of cytokines and their receptors are shown in Figure 3.

Despite the importance of these mechanisms in influencing protein expression and function, to the best of our knowledge, fewer studies regarding cytokine signaling have been performed in Chagas disease. It has been shown that STAT4 is associated with heart failure in patients with dilated cardiomyopathy [31]. Furthermore, it has been described that *T. cruzi* can release extracellular vesicles that act on macrophages activating the NF-kB signaling pathway, which in turn, produces inflammatory cytokines contributing to cardiac damage and exacerbated inflammatory pathology observed in CCC [168]. In addition, the blockade of this pathway is important for the reduction of cardiac damage [169] and may emerge as a potential target for intervention. Therefore, analysis of the expression of cytokine receptors and activated STATs will also provide important information regarding cytokine-mediated immune control and unveil additional targets.

## 5. Concluding Remarks

The significance of this review is that it provides a comprehensive assessment of the role of cytokine and immunoregulation in the differential clinical evolution of human Chagas disease, presenting critical aspects related to their role in controlling or exacerbating the inflammatory response, as well as pre- and post-transcriptional control of these molecules. Below, we highlight some of the potential future directions that can arise from the current knowledge in the area. There is convincing evidence that cytokine networks can influence the clinical outcome of CD. In fact, the studies reviewed in this article reinforce the correlation of inflammatory cytokines, such as IFN-gamma and TNF, with the worst clinical outcome and progression of CCC. Cytokines with a modulatory profile are correlated with the maintenance of IND, without impairment of cardiac and digestive functions. However, the mechanisms by which these molecules act, as well as the complex signaling pathways activated by cytokines, are not completely understood. Additionally, there are a few studies of SNPs in cytokine coding genes in CD patients. Despite that, signaling pathways and SNPs certainly have an impact on the clinical course of the disease. It is important to emphasize that several other aspects of the host, as well as the parasite’s intrinsic characteristics, are key determinants in cytokine responses. In addition, although many studies assess the profile of cytokines in individuals with established chronic disease, longitudinal study cohorts that follow the profile of this immune network from the beginning of the infection should be encouraged to understand the importance and impact of these molecules in CD evolution. Given the solid and extensive knowledge gained over the years related to cytokine influence in Chagas disease severity, future studies focused on their modulation, or on their receptors and signaling pathways, to control disease pathology may provide important immunotherapeutic alternatives to treat Chagas disease alone or in combination with anti-parasite drugs.

## Figures and Tables

**Table 1:** Cytokines, chemokines, and cytotoxic molecules associated with distinct clinical outcomes of human Chagas disease.

**Cytokines and Their Receptors**	**Role**	**Source**	**References**
**Association with** **the Development of CCC**			
IFN-gamma	Inflammation, T-cell activation	PBMC, heart	[22,24,29,30,37,38,39]
TNF	Inflammation, T-cell activation, worse cardiac function	Plasma, blood, PBMC, heart	[18,28,31,32,38,40,41,42]
sTNFR1 and sTNFR2	Inflammation, worse cardiac function	Plasma	[43]
IL-6	Inflammation, worse cardiac function	Plasma	[30,44,45]
IL-1β	Inflammation	Plasma	[30,46]
TGF-β	Tissue fibrosis	Serum, heart	[47]
MIF	Inflammation, progression	Serum	[48]
**Association with protection to CCC**			
IL-2	Low in CCC	PBMC	[49]
IL-10	Protective immune response, better cardiac function	Plasma, PBMC, whole blood	[18,30,38,39,40,41,50]
IL-17	Protective immune response, better cardiac function	Plasma, PBMC	[38,51,52,53]
**Fibrotic and Cytotoxic Molecules**	**Description**	**Source**	**References**
**Association with** **the Development of CCC**			
MMP-2	Tissue fibrosis	Plasma	[54,55]
MMP-9	Tissue fibrosis	Plasma, PBMC	[54,56]
MMP-2/MMP-9 ratio	Pathological cardiac remodeling	Plasma, heart	[57,58]
Fibronectin	Recruitment of inflammatory T cells to the heart	Heart	[42]
Granzyme A	Myocyte dysfunction	Heart	[28]
**Chemokines and Their Receptors**	**Description**	**Source**	**References**
**Association with** **the Development of CCC**			
CXCL9	Recruitment of inflammatory cells	Plasma, heart	[46,59,60]
CXCL10	Recruitment of inflammatory cells	Plasma	[46,61,62]
CCL5	Recruitment of inflammatory cells	Serum	[63]
CCL2	Recruitment of inflammatory cells	Serum and plasma	[32,63]
CCR5	Recruitment of inflammatory cells, Worse prognosis	PBMC	[62,64]
CXCR3	Recruitment of inflammatory cells	PBMC	[64]

**Table 2:** Polymorphisms in genes associated with the immune response and their association with distinct clinical outcomes of human Chagas disease.

**Gene Polymorphism**	**Description**	**References**
***Association with Development of CCC***		
*IL4RA*	Interleukin 4 receptor	[101]
*IKBL/NFKBIL1*	NF-kappa-B inhibitor-like protein 1	[102]
*IL12ß; IL12*	Pro-inflammatory cytokine	[99,103]
*IL17A; IL17F*	Protective cytokine	[104,105,106]
*NLRP1*	Protein involved in inflammasome	[107]
*CASP1*	Protein involved in inflammatory cascade	[108]
*Lymphotoxin*	Member of the TNF superfamily of cytokines; are responsible for regulating the growth and function of lymphocytes	[109,110]
*PI3 kgamma*	Molecules involved in signaling pathway of the efficient immune response against *T. cruzi*	[111]
***Association with Protection to CCC***		
*CXCL10, CCL5, CXCL9*	Chemokine Ligand	[60,63]
*CTLA-4*	Cytotoxic T-lymphocyte-associated antigen 4	[112]
*VPAC1*	Vasoactive intestinal peptide (VIP) receptors 1	[113]
***No association with CCC***		
*IL4*	Anti-inflammatory cytokine	[101,103,114]
*MIF*	Macrophage migration inhibitory factor	[115]
*IL1A, IL6*	Pro-inflammatory cytokine	[103,116]
*TGF-β1*	Multifunctional cytokine	[99,117,118]
*TLR1, TLR2 TLR4, TLR6*	Toll-like receptor (TLR)	[119,120]
*TNFR1; TNFR2;*	Tumor necrosis factor receptor	[99]
*Galectin-3*	Member of the lectin family/ cell–cell adhesion	[121]
*CARD11*	Protein involved in the function of immune system cells	[107]
*FOXP3*	Protein involved in immune system responses	[122]
***Variable According to the Population Studied***		
*MHC genes*	Major histocompatibility complex/presentation of internal or external antigens to the T cells	[123,124,125,126]
*CCR5; CCR2*	Chemokine receptor type	[60,109,127,128,129,130]
*TNFA; TNFB; IL1B; IFN-g*	Pro-inflammatory cytokine	[99,103,131,132,133,134,135,136,137,138]
*IL10*	Anti-inflammatory cytokine	[97,99,114]
*IL1RN*	Interleukin-1 receptor antagonist	[131,132]
*MAL/TIRAP*	Encodes an adaptor protein for TLR	[120,139,140]
*BAT-1*	Anti-inflammatory activity associated with reduced expression of HLA-B-1	[99,141]
*CCL2/MCP-1*	Chemokine ligand 2	[63,142]

## Data Availability

Not applicable.
